# Large, Life-Threatening Right Ventricular and Pulmonary Artery Thrombus Revealing Antiphospholipid Syndrome: The Role of Risk Stratification and Thrombolytic Therapy

**DOI:** 10.7759/cureus.116979

**Published:** 2026-09-26

**Authors:** Hamza Seghrouchni, Zineb Boubekri, Hiba Haouani, Fadoua Achour, Zayna Nadhil, Nadia Fellat

**Affiliations:** 1 Cardiology A Department, Centre Hospitalier Ibn Sina, Rabat, MAR; 2 Cardiology, Faculty of Medicine and Pharmacy of Rabat, Rabat, MAR

**Keywords:** antiphospholipid syndrome, pulmonary embolism, right ventricular thrombus, tenecteplase, thrombolysis

## Abstract

Right ventricular thrombus in the setting of acute pulmonary embolism is an uncommon and potentially life-threatening finding associated with a high risk of hemodynamic deterioration. The optimal management remains controversial, particularly in patients with an extensive thrombotic burden but without overt hemodynamic instability. We report a case of a patient presenting with an extensive thrombus occupying the right ventricle and extending into the pulmonary artery. Multimodality imaging demonstrated a significant thrombotic burden with right ventricular involvement and confirmed acute pulmonary embolism. Echocardiographic assessment revealed right ventricular thrombus and signs of right ventricular pressure overload. Based on the clinical presentation, imaging findings, and right ventricular involvement, the patient was classified as having intermediate-high-risk pulmonary embolism. Given the large mobile right ventricular thrombus with pulmonary arterial extension, profound hypoxemia (oxygen saturation: 54%) on room air, and severe tachypnea (45 breaths/min), systemic thrombolysis was considered despite the absence of hemodynamic collapse. After multidisciplinary discussion, an individualized treatment decision was made to administer reduced-dose tenecteplase (0.25 mg/kg; 15 mg). Marked clinical improvement was observed within 24 hours, with no major bleeding complications. Further investigations revealed antiphospholipid syndrome as the underlying prothrombotic condition. At three-month follow-up, the patient remained clinically stable, with a marked reduction in the residual right ventricular thrombus and resolution of the pulmonary trunk thrombus. This case highlights the importance of early echocardiographic identification of right ventricular thrombus in patients with pulmonary embolism and the role of multimodality imaging in therapeutic decision-making. It also illustrates the potential role of reduced-dose systemic thrombolysis in selected patients with intermediate-high-risk pulmonary embolism and extensive intracardiac thrombotic burden. Recognition of the underlying prothrombotic disorder is essential for guiding long-term anticoagulation and preventing recurrent thromboembolic events.

## Introduction

Pulmonary embolism (PE) is a major cardiovascular condition caused by obstruction of the pulmonary arterial circulation, most commonly by thromboembolic material [1]. Its estimated worldwide incidence ranges from 39 to 115 cases per 100,000 individuals annually, making PE an important cause of cardiovascular morbidity and mortality [2-4].

PE is frequently associated with deep vein thrombosis (DVT) of the lower limbs, and together they constitute venous thromboembolism (VTE). Beyond anticoagulation and thrombolytic therapy, identifying underlying prothrombotic conditions is important to guide long-term management and reduce the risk of recurrence. Among these, antiphospholipid syndrome (APS), a rare autoimmune disorder, is an important acquired risk factor for DVT [5], and PE may be the first manifestation of APS and represents an important cause of morbidity and mortality in APS, underscoring the importance of early recognition and adequate therapy.

The management of PE becomes particularly challenging when a right ventricular (RV) thrombus is present. Although an RV thrombus may reflect a substantial thrombotic burden and be associated with RV dysfunction, the optimal management of patients with RV thrombus and PE who remain normotensive is not clearly established. Systemic thrombolysis may be considered in selected patients with severe clinical or RV compromise, but its potential benefits must be weighed against the risk of major bleeding.

We present a case of extensive bilateral PE associated with a large mobile RV thrombus and significant RV dysfunction in a normotensive patient with severe respiratory compromise and subsequently identified APS. Reduced-dose tenecteplase was administered after multidisciplinary discussion. This case highlights the therapeutic dilemma of thrombolysis in normotensive PE with substantial thrombus burden and RV dysfunction, and discusses the rationale and limitations of using a reduced-dose tenecteplase regimen in this setting.

## Case presentation

We report a case of a 27-year-old female with no cardiovascular risk factors. She had three pregnancies, of which only one reached term. She had been taking oral contraceptives for the past two years.

One week before admission, the patient developed sudden-onset dyspnea classified as New York Heart Association (NYHA) functional class III [6]. She initially sought medical attention at a nearby health center, where she was prescribed anxiolytics and a beta-blocker and subsequently discharged home. Later that night, her dyspnea worsened, prompting her to seek care at the emergency department of the university hospital. On admission, the patient presented with severe respiratory distress, with a respiratory rate of 45 breaths/min and a peripheral oxygen saturation (SpO₂) of 54% on room air. Clinical examination revealed perioral cyanosis and cold distal extremities, without signs of right heart failure or cardiac murmur; the pulmonary fields were clear, and there was no infectious history. Blood pressure was 125/72 mmHg, and neurological status was preserved. Non-invasive ventilation was initiated with a fraction of inspired oxygen (FiO₂) of 60%, resulting in an improvement in SpO₂ to 93%. Arterial blood gas analysis performed under oxygen therapy showed a pH of 7.42, partial pressure of carbon dioxide (PaCO₂) of 23 mmHg, partial pressure of oxygen (PaO₂) of 66 mmHg, and lactate of 0.9 mmol/L.

ECG showed sinus tachycardia at 120 bpm, a left-axis deviation, incomplete right bundle branch block associated with a left anterior fascicular block, and inverted T waves in the anterior and inferior leads (Figure 1).

**Figure 1:**
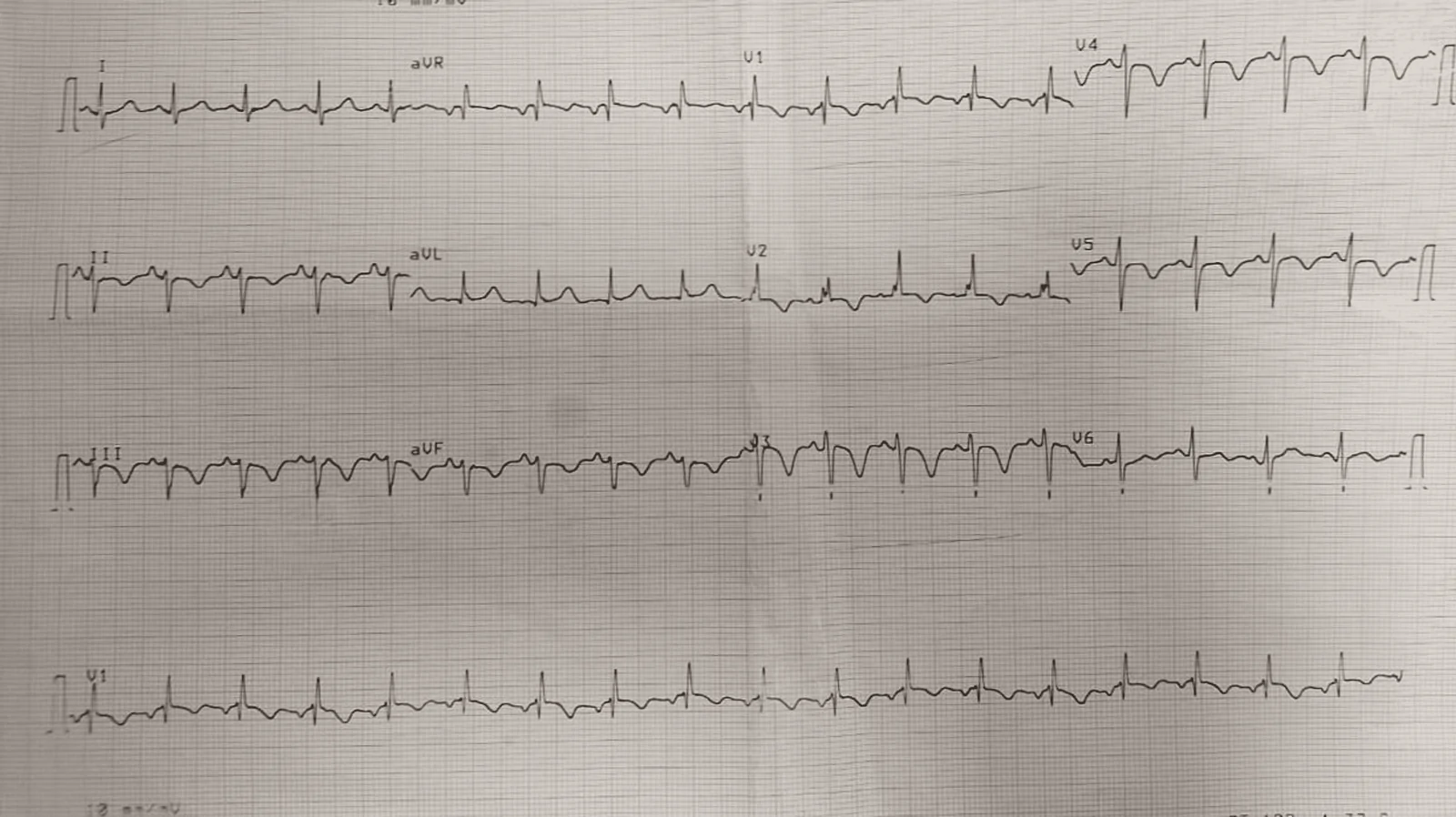
Twelve-lead ECG performed at the emergency department.

PE was suspected first with intermediate probability as per the revised Geneva score [7]. Laboratory tests showed elevated D-dimers. CT pulmonary angiography confirmed massive bilateral proximal PE that evolved to pulmonary infarction with a large, threatening RV thrombus (Figure 2).

**Figure 2:**
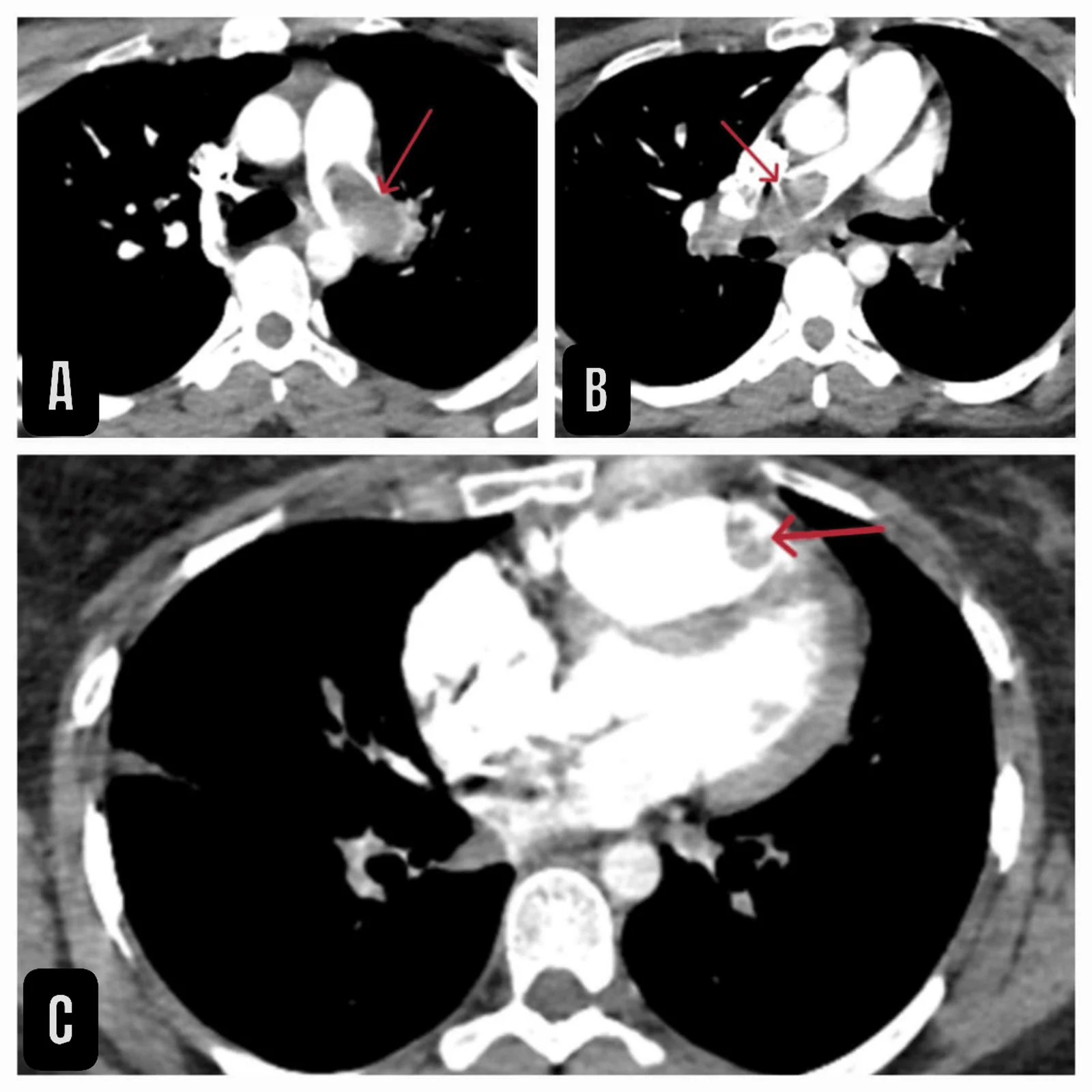
Computed tomography pulmonary angiography demonstrating extensive pulmonary embolism with an associated right ventricular thrombus. (A) Axial image showing a thrombus in the main pulmonary artery. (B) Axial image showing a thrombus in the right pulmonary artery. (C) Axial image showing a large intraventricular thrombus in the right ventricle. Red arrows indicate the thrombi.

Anticoagulation with unfractionated heparin (UFH) was initiated with an 80 IU/kg intravenous bolus followed by continuous infusion at 500 IU/kg/day using an auto-pulse syringe, with activated partial thromboplastin time (aPTT) monitoring every four hours and a target ratio of 2-3. UFH infusion was discontinued before systemic thrombolysis with tenecteplase and resumed after thrombolysis based on the first post-thrombolysis aPTT, which was 63 seconds. The simplified Pulmonary Embolism Severity Index (sPESI) score was 2 [8].

Bedside transthoracic echocardiography (TTE) revealed acute cor pulmonale, with a moderately dilated right ventricle containing a large thrombus measuring 4.7 × 2.6 cm (Figure 3 and Video 1). Right ventricular systolic function was mildly impaired, with a tricuspid annular systolic velocity on tissue Doppler (S′) of 9 cm/s and a tricuspid annular plane systolic excursion (TAPSE) of 14 mm, indicating impaired longitudinal RV systolic function. A right ventricular/left ventricular (RV/LV) ratio >1 (Figure 4) and paradoxical septal motion with a D-shaped left ventricle were consistent with acute RV pressure overload. The right atrium was borderline enlarged and contained microthrombi. Pulmonary acceleration time was markedly shortened at 38 ms, consistent with elevated pulmonary vascular pressure, while estimated pulmonary artery systolic pressure was 54 mmHg, with findings meeting the 60/60 sign. The pulmonary trunk was dilated with endoluminal thrombus (Figure 5). The left ventricle was hyperkinetic with a slightly reduced subaortic velocity-time integral (VTI) of 11 cm.

**Figure 3:**
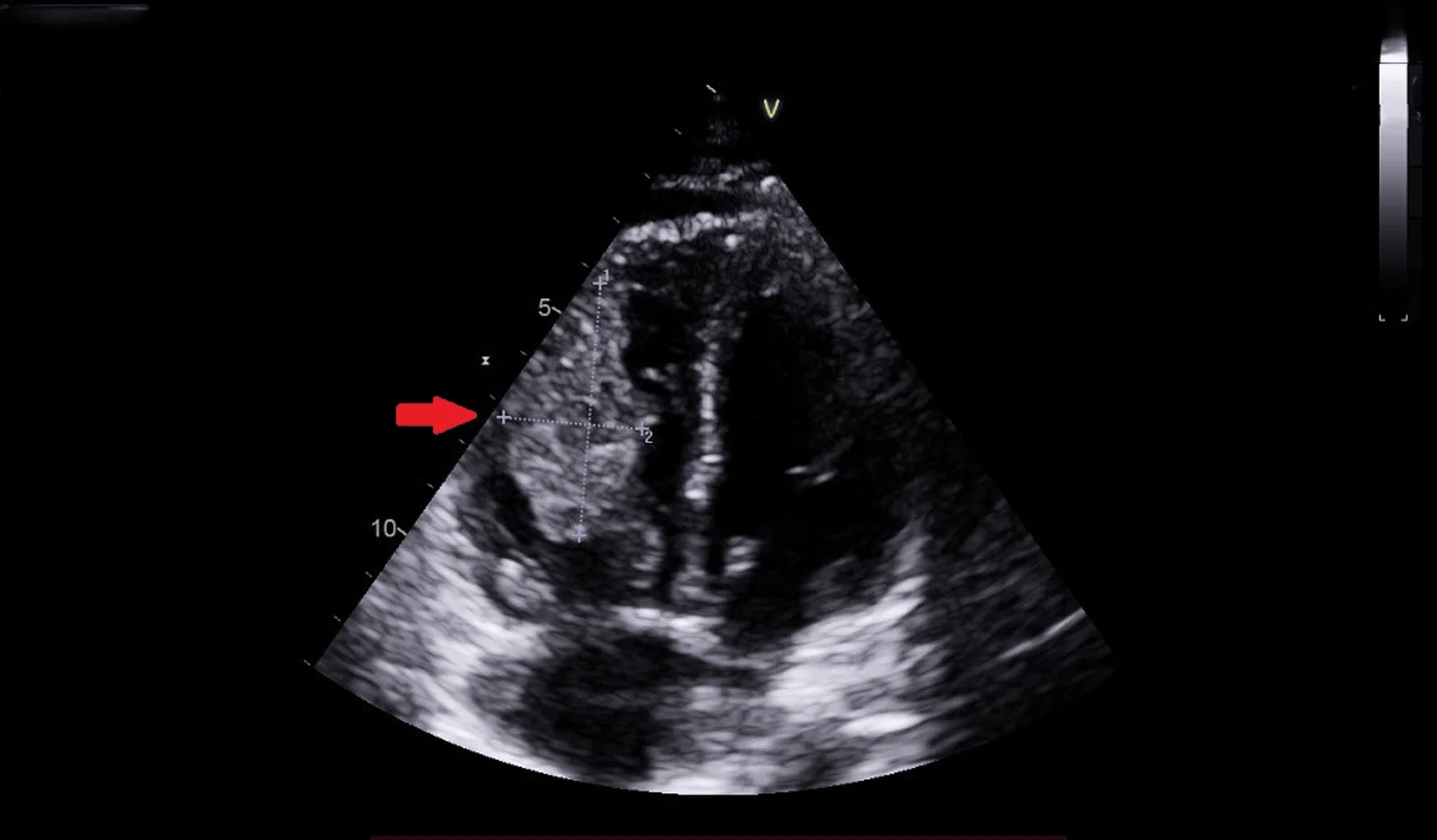
Transthoracic echocardiography at initial presentation. Apical four-chamber view showing a large right ventricular thrombus measuring 4.7 × 2.6 centimeters. The red arrow indicates the thrombus within the right ventricle.

**Video 1:** Transthoracic echocardiography at initial presentation. Apical four-chamber view showing a large right ventricular intraventricular thrombus.

**Figure 4:**
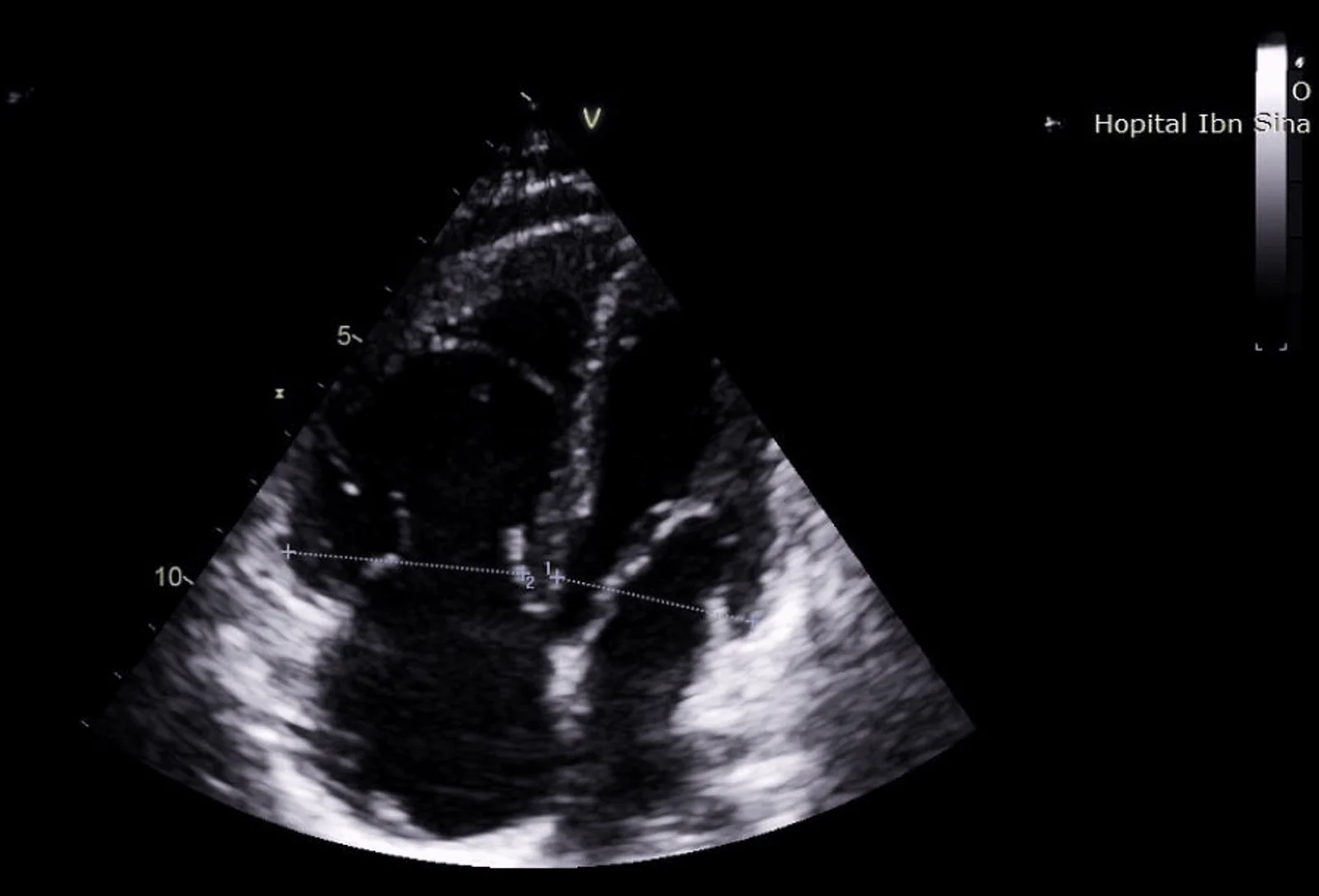
Transthoracic echocardiography at initial presentation. Apical four-chamber view showing right ventricular dilatation with an end-diastolic RV/LV ratio greater than 1. RV: right ventricle; LV: left ventricle.

**Figure 5:**
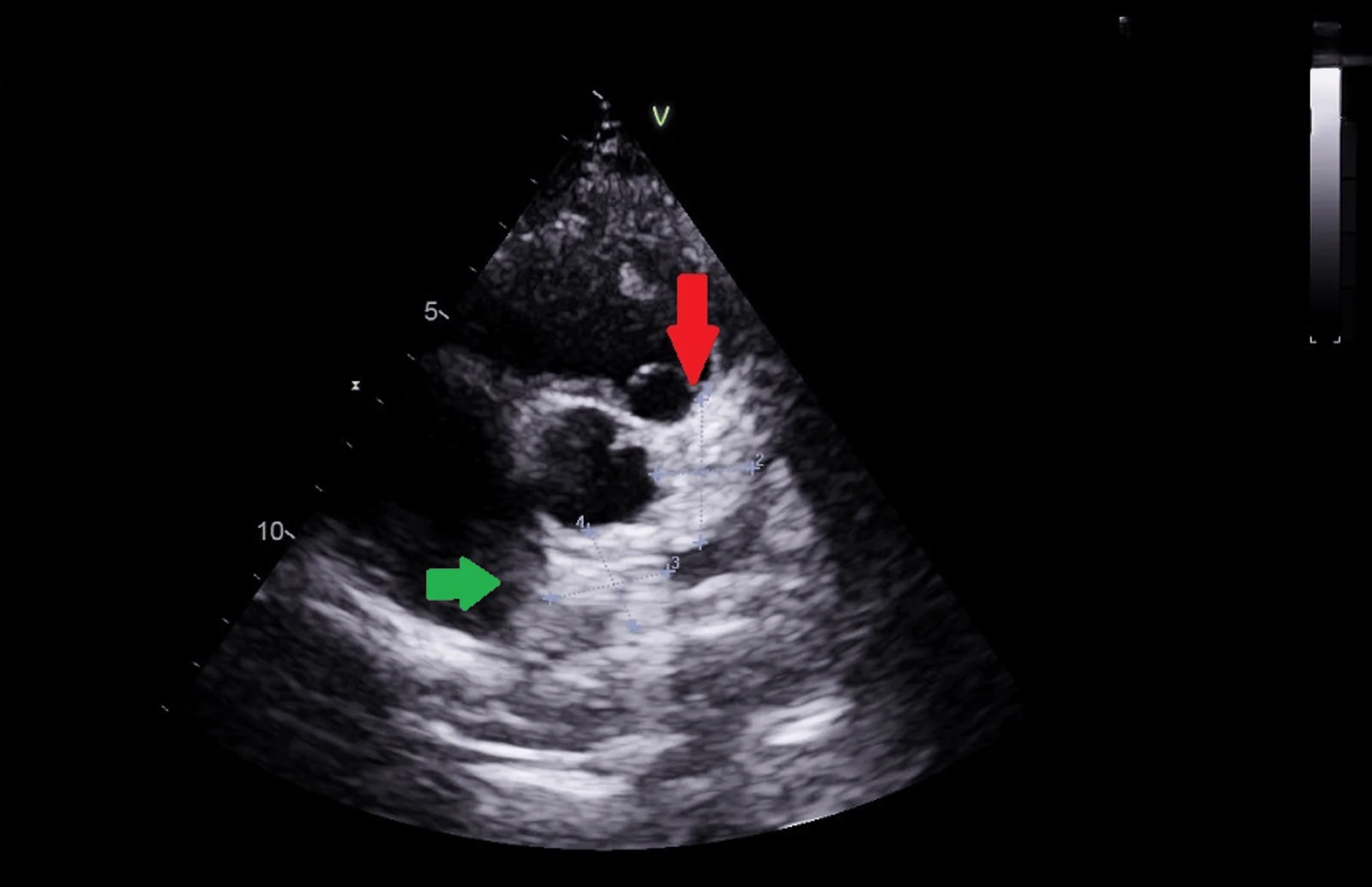
Transthoracic echocardiography at initial presentation. Parasternal short-axis view at the level of the great vessels showing thrombi in the main pulmonary artery, measuring 2.7 × 1.8 centimeters, and in the right pulmonary artery, measuring 2.3 × 2.0 centimeters. The red arrow indicates the thrombus in the main pulmonary artery, while the green arrow indicates the thrombus in the right pulmonary artery.

N-terminal pro-B-type natriuretic peptide (NT-proBNP) was markedly elevated at 11,371 pg/mL. No high-sensitivity troponin measurement was performed at admission; however, a positive high-sensitivity troponin result of 0.5 ng/mL (female reference value <0.016 ng/mL) was subsequently identified on day 2 of hospitalization during a review of the electronic medical record. In the context of documented RV dysfunction and elevated cardiac biomarkers, the patient was subsequently classified as intermediate-high risk according to the 2019 European Society of Cardiology (ESC) risk stratification framework [9].

Given the severe respiratory compromise requiring non-invasive ventilation, extensive bilateral proximal PE with pulmonary trunk obstruction, a large mobile RV thrombus, and significant RV dysfunction, systemic thrombolysis was considered after multidisciplinary discussion despite preserved blood pressure. The patient weighed 60 kg and received reduced-dose tenecteplase (0.25 mg/kg; total dose 15 mg). No formal bleeding-risk score was calculated; clinical assessment identified a young patient without a history of bleeding and with preserved renal function. The reduced dose was chosen to balance the need for rapid thrombus reduction against the bleeding risk associated with systemic thrombolysis, in the context of limited and heterogeneous evidence regarding the optimal tenecteplase dose for acute pulmonary embolism.

The patient's clinical and hemodynamic condition improved within 24 hours, with resolution of respiratory distress and reduced oxygen requirements. Follow-up TTE demonstrated a marked reduction in the size of the RV and pulmonary artery thrombi.

The initial lupus anticoagulant (LA) assessment was positive. Blood sampling was performed 13 hours after the last dose of enoxaparin to minimize potential anticoagulant interference. Following this assessment, anticoagulation was transitioned to acenocoumarol with enoxaparin bridging until the target international normalized ratio (INR) of 2-3 was achieved, after which enoxaparin was discontinued. Repeat testing performed 12 weeks later demonstrated persistent LA positivity, with a positive normalized LA1/LA2 ratio, while anticardiolipin antibodies (aCL) and anti-β2 glycoprotein I (aβ2GP1) antibodies remained negative (Table 1). For the repeat assessment, acenocoumarol was temporarily discontinued, and the INR was 1.12 at the time of blood sampling; anticoagulation was subsequently resumed with enoxaparin bridging and acenocoumarol until the target INR of 2-3 was achieved, after which enoxaparin was discontinued. In the presence of objectively confirmed pulmonary embolism, a history of pregnancy losses, and persistent LA positivity, the diagnosis of APS was established in consultation with the internal medicine team, according to the revised Sapporo/Sydney classification criteria [10]. Lifelong anticoagulation with acenocoumarol was planned. The patient showed marked clinical improvement and was classified as NYHA functional class I [6]. TTE showed a normal-sized RV with preserved longitudinal systolic function and a complete resolution of the thrombus in the pulmonary trunk (Figure 6). A small, organized residual thrombus measuring 0.88 × 0.66 cm remained in the right ventricle (Figure 7). Pulmonary hypertension was evaluated with a tricuspid regurgitation velocity (TRV) of around 3.34 m/s.

**Table 1:** Serial antiphospholipid antibody testing demonstrating persistent lupus anticoagulant positivity at three months. LA: lupus anticoagulant; LA1: lupus anticoagulant screening test; LA2: lupus anticoagulant confirmatory test; aCL: anticardiolipin antibodies; aβ2GPI: anti-β2-glycoprotein I antibodies; GPLU: IgG phospholipid units; MPLU: IgM phospholipid units.

	Initial values	Values after 3 months	Reference ranges	Interpretation
LA1	1.57	1.51	<1.2	Positive LA
LA2	1.1	1.1	<1.2	
Normalized LA1/LA2 ratio	1.43	1.37	>1.2	
Anticardiolipin antibodies IgG	<3 GPLU/ml	<3 GPLU/ml	<12 GPLU/ml	Negative aCL
Anticardiolipin antibodies IgM	<3 MPLU/ml	<3 MPLU/ml	<12 MPLU/ml	
Anti-β2-glycoprotein I antibodies IgG	1.5 U/ml	1 U/ml	<8 U/ml	Negative aβ2GP1
Anti-β2-glycoprotein I antibodies IgM	1 MPLU/ml	1 MPLU/ml	<12 MPLU/ml	

**Figure 6:**
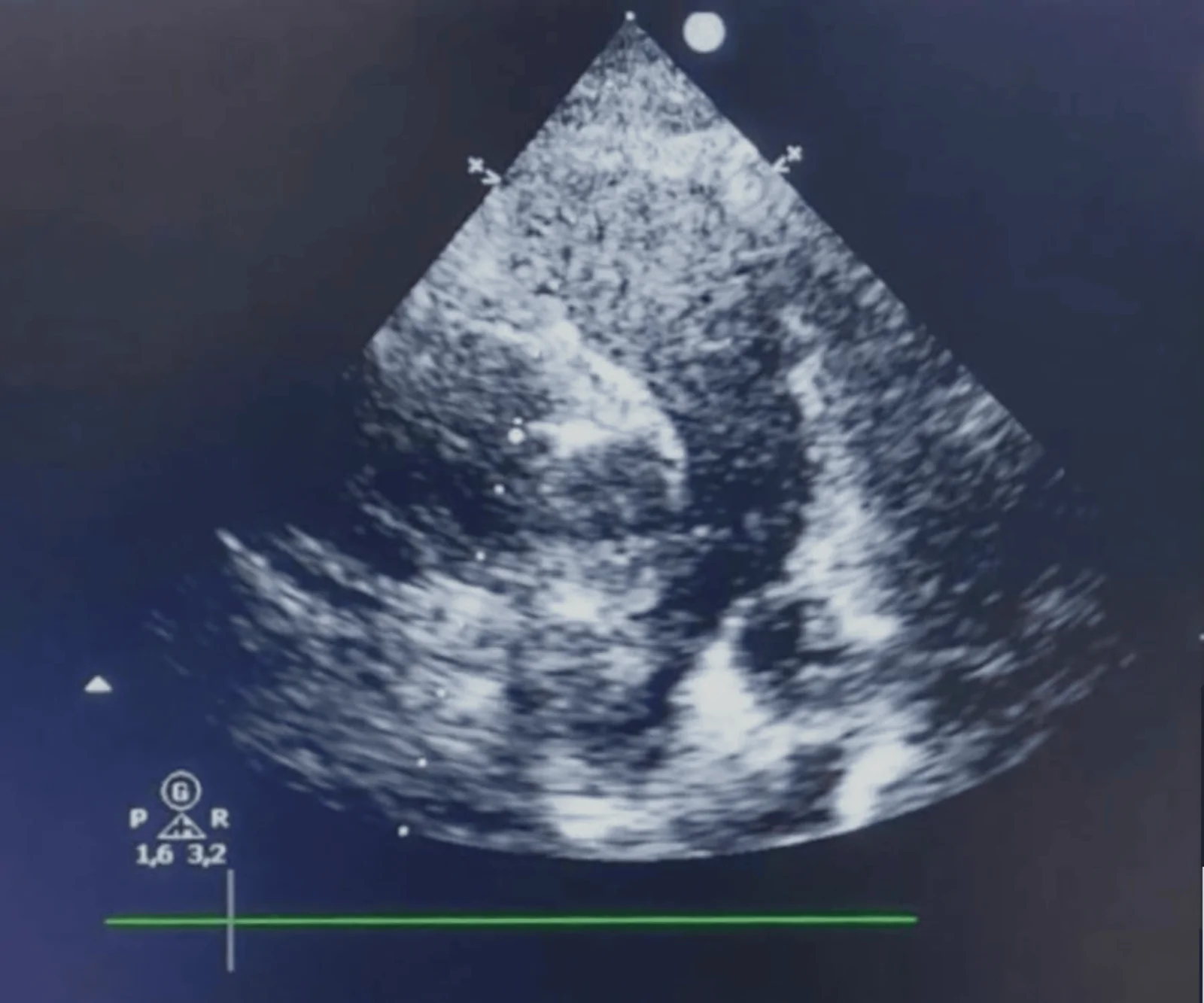
Transthoracic echocardiography at three-month follow-up showing no visible residual thrombus in the pulmonary trunk on the parasternal short-axis view at the level of the great vessels.

**Figure 7:**
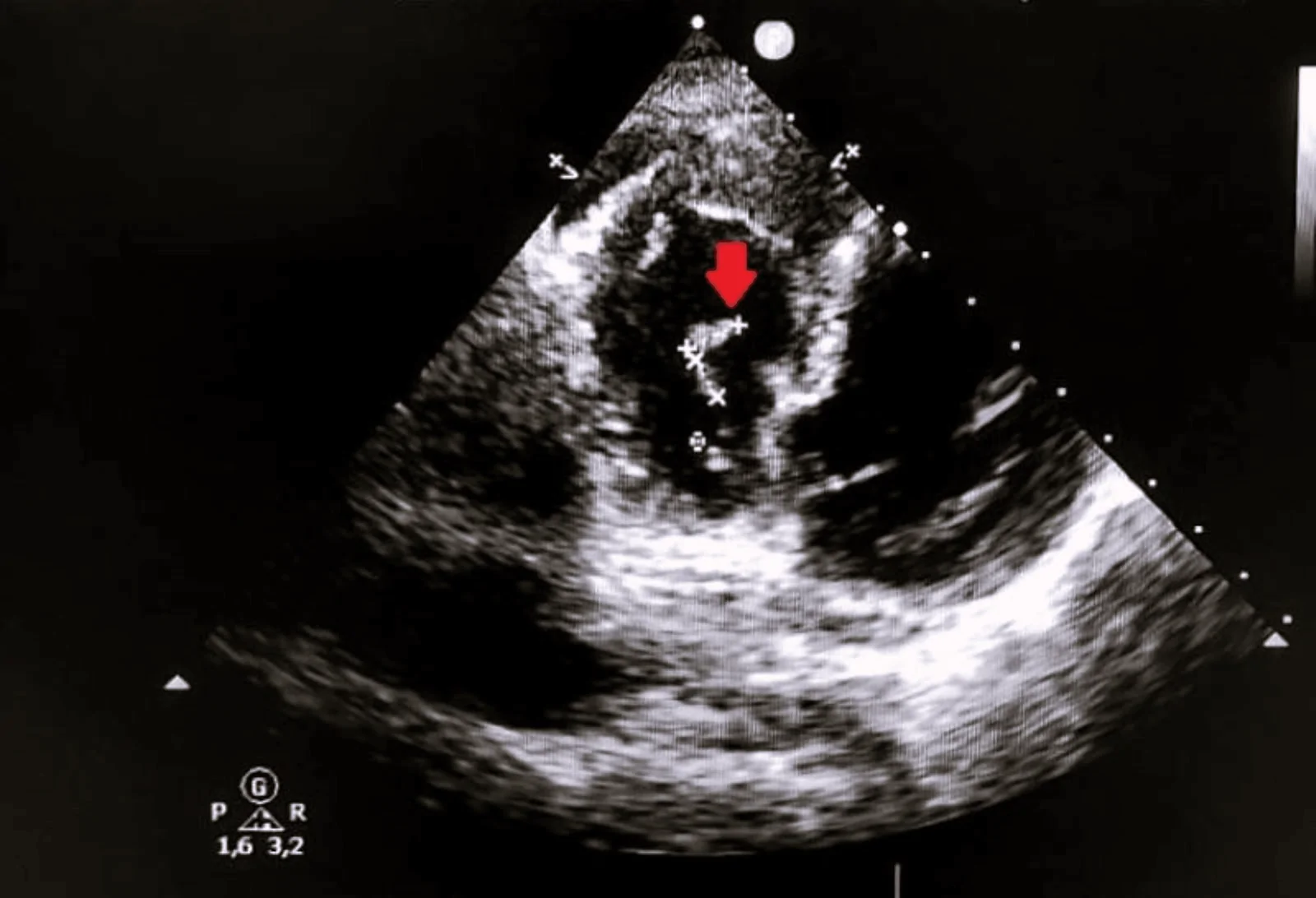
Transthoracic echocardiography at three-month follow-up showing a small residual thrombus in the right ventricle on the parasternal short-axis view at the level of the right ventricle, measuring 0.88 × 0.66 cm. The red arrow indicates the residual right ventricular thrombus.

## Discussion

APS-related PE and RV thrombus

APS is an autoimmune disorder characterized by thrombotic events, venous, arterial, or microvascular and/or obstetric morbidity occurring in the presence of persistently positive antiphospholipid antibodies (aPL), including lupus anticoagulant (LA), IgG and/or IgM anti-β2 glycoprotein I (aβ2GP1), and anticardiolipin antibodies (aCL) [11]. The diagnosis requires at least one documented episode of vascular thrombosis or recurrent pregnancy morbidity in association with positive aPL tests performed on two or more occasions, at least 12 weeks apart [12]. APS may coexist with other autoimmune diseases and is reported in approximately 7-15% of patients with systemic lupus erythematosus (SLE). Moreover, aPL are detected in 1-5 % of the general population, although only a minority develop clinical manifestations of the syndrome [11].

Beyond atherosclerosis-related cardiovascular disease, valvular abnormalities, particularly leaflet thickening and sterile vegetations, represent the most frequent cardiac involvement in APS [12]. Other cardiovascular complications include coronary artery disease, myocardial dysfunction, pulmonary hypertension, and intracardiac thrombus formation [12]. The pathogenesis is thought to result from both immune-mediated injury and thrombotic mechanisms. According to the “two-hit” hypothesis, persistent aPL generate a chronic prothrombotic milieu (first hit), whereas an additional trigger such as infection, inflammation, pregnancy, surgery, or cessation of anticoagulation precipitates overt thrombosis (second hit). PE, a common manifestation of VTE, often occurs under these conditions. The principal prothrombotic mechanisms mediated by aPL include endothelial dysfunction, platelet activation, impaired fibrinolysis, and complement activation [13].

The thrombotic phenotype of APS is determined by the interplay between the patient’s antiphospholipid antibody profile and additional clinical or environmental risk factors. Patients with persistent triple-positive serology, involving LA, aCL, and anti-β2GPI, are considered to have the highest thromboembolic risk, whereas isolated low-titer positivity for a single antibody is associated with a lower, although not negligible risk. Previous thromboembolic events, including DVT or PE, are particularly important predictors of recurrence. Concomitant inherited thrombophilias, such as factor V Leiden, the prothrombin mutation, or protein C or S deficiency, may further contribute to thrombotic risk and can be relevant when considering long-term secondary prevention. In women with APS, pregnancy, menopause, hormone replacement therapy, and combined oral contraceptive use may further increase thrombotic susceptibility. Other conditions, including obesity, dyslipidemia, hypertension, and diabetes mellitus, may also amplify the prothrombotic state through endothelial dysfunction, chronic low-grade inflammation, and platelet activation, contributing to both venous and arterial thrombotic manifestations. [13].

In a literature review published in 2016 by Dhibar et al. [14], 28 indexed published cases of APS with intracardiac thrombus between 1989 and 2012 were identified using a MEDLINE search with MeSH terms such as "APS and intra-cardiac thrombus". They found that 25% of the cases had an isolated RV thrombus or were associated with other cardiac chamber thrombus; 67% of the cases presented with PE. Only three of the cases had concomitant RV thrombus, which highlights the rarity and clinical significance of this association.

Tenecteplase in intermediate-high-risk pulmonary embolism

According to the European Society of Cardiology/European Respiratory Society 2019 guidelines, patients with acute PE are stratified into high-, intermediate-, and low-risk categories. Risk stratification plays a central role in guiding the management of PE, as also supported by real-world studies [9].

The most recent American guidelines by the American Heart Association (AHA)/American College of Cardiology (ACC) on PE management describe five categories to classify PE risk ranging from category A (subclinical PE) to category E (PE with cardiopulmonary failure), adding a “Respiratory modifier” to these risk categories [15]. Our patient presentation falls under category CR, which corresponds to symptomatic PE with an elevated clinical severity score and respiratory distress, according to these new outlines. Management would be anticoagulation, and thrombolysis is not recommended based on these 2026 guidelines. However, in our case, the associated RV thrombus influenced the management decision, and an unconventional out-of-guideline decision of thrombolysis allowed for patient stabilization.

The primary therapeutic objectives in acute PE, particularly in intermediate-high and high-risk patients, are the restoration of pulmonary blood flow through reperfusion, the reduction of right ventricular dysfunction, and the prevention of recurrent PE, thereby stabilizing hemodynamics [1].

Conventional management primarily relies on anticoagulation therapy, including parenteral agents such as low-molecular-weight heparin (LMWH) or UFH, as well as direct oral anticoagulants (DOACs). Compared with anticoagulation alone, thrombolytic therapy has been shown to improve RV wall motion within 24 hours of treatment initiation [1].

Evidence indicates that in high-risk PE, thrombolytic therapy significantly reduces both mortality and recurrence rates. However, its use in intermediate-risk PE remains controversial due to the balance between potential clinical benefit and the increased risk of bleeding [1].

Thrombolytic therapy is associated with an increased risk of bleeding complications. In the PEITHO trial, fibrinolytic therapy in patients with intermediate-risk PE was associated with a 2.0% incidence of hemorrhagic stroke and a 6.3% rate of major extracranial bleeding [16].

Tenecteplase, a genetically engineered variant of alteplase, has demonstrated therapeutic potential in the management of stroke and cardiovascular disease. Compared with conventional thrombolytic agents such as alteplase, which requires a two-hour intravenous infusion, tenecteplase offers the advantage of administration as a single intravenous bolus, thereby simplifying and accelerating treatment delivery [1].

A meta-analysis conducted by Zhang et al. [1] included four RCTs and two cohort studies aiming to assess the efficacy of tenecteplase in patients with high and intermediate high-risk PE. Four RCTs suggested that tenecteplase in patients with intermediate-high-risk PE reduced RV insufficiency at 24 hours after treatment and the incidence of hemodynamic failure, without affecting mortality in the short or long term.

Tenecteplase may reduce the risk of disease progression and improve clinical outcomes in selected patients with intermediate-risk PE. In the PEITHO study, the largest randomized, placebo-controlled trial of fibrinolysis in intermediate-risk PE to date, tenecteplase was associated with a reduction in hemodynamic decompensation within seven days [16]. However, this potential benefit must be balanced against the risk of bleeding associated with systemic thrombolysis.

The bleeding risk associated with tenecteplase has varied across studies. Becattini et al. [17] reported no significant increase in major bleeding with tenecteplase, whereas the PEITHO trial reported an increased risk of major bleeding, including intracranial hemorrhage, within seven days [16]. These findings highlight the importance of careful patient selection and individualized assessment of the benefit-risk balance before systemic thrombolysis.

Overall, the meta-analysis by Zhang et al. [1] concluded that tenecteplase was associated with a higher risk of bleeding within seven days in patients with intermediate-risk PE, without a significant effect on long-term bleeding events.

The doses of tenecteplase used in this meta-analysis were 0.5 mg/kg [1]. Evidence from other settings suggests that reduced-dose thrombolytic regimens may be associated with a lower risk of bleeding. In a meta-analysis conducted by Xu et al. [18] evaluating the efficacy and safety of tenecteplase versus alteplase in acute ischemic stroke, half-dose tenecteplase (0.25 mg/kg) was associated with early neurological improvement and a lower risk of intracranial hemorrhage compared with alteplase. However, these findings are derived from the stroke population and therefore provide only indirect support for the use of reduced-dose tenecteplase in PE.

Similarly, a prospective randomized multicenter trial including 118 patients with PE and hemodynamic instability or massive pulmonary artery obstruction compared 50 mg/2 h with 100 mg/2 h of alteplase. The 50 mg regimen showed similar efficacy and a more favorable safety profile compared with the 100 mg regimen in acute PE [19]. Although these findings support the concept that reduced-dose thrombolysis may offer a potential safety advantage in PE, they cannot be directly extrapolated to tenecteplase. Thus, these studies provide an indirect rationale for considering a reduced-dose tenecteplase regimen of 0.25 mg/kg in selected patients with PE, but do not establish its efficacy or safety in this setting.

## Conclusions

PE is a recognized complication and a major cause of morbidity and mortality in patients with APS. In this young patient, PE occurred in the setting of oral contraceptive use and previously unrecognized APS, highlighting the importance of considering underlying thrombophilia in young patients presenting with venous thromboembolism.

The patient presented with intermediate-high-risk PE associated with a mobile right ventricular thrombus and respiratory distress and was treated with reduced-dose systemic tenecteplase, with subsequent clinical improvement and no major bleeding observed. The observed outcome in this case supports the need for further evaluation of thrombolytic strategies in selected patients with intermediate-high-risk PE and associated right ventricular thrombus.
